# Bridge Damage Identification Using Time-Varying Filtering-Based Empirical Mode Decomposition and Pre-Trained Convolutional Neural Networks

**DOI:** 10.3390/s25154869

**Published:** 2025-08-07

**Authors:** Shenghuan Zeng, Jian Cui, Ding Luo, Naiwei Lu

**Affiliations:** 1 Shenzhen Expressway Engineering Testing Co., Ltd., Shenzhen 518000, China; zengshenghuan@szewec.com (S.Z.); luoding@szewec.com (D.L.); 2 School of Civil Engineering, Changsha University of Science and Technology, Changsha 410114, China; cuijian@csust.edu.cn

**Keywords:** structural damage identification, structural health monitoring, time-frequency analysis, signal processing, convolutional neural network

## Abstract

**Highlights:**

**What are the main findings?**

- A novel bridge damage identification framework is proposed, combining TVFEMD for signal denoising and pre-trained CNNs for accurate damage classification.
- The study finds that ResNet-50 performs optimally in damage classification tasks, especially when processing TVFEMD-processed signals, with improved clustering and separability of features.

**What is the implication of the main finding?**

- The proposed method improves the robustness of structural health monitoring systems in noisy environments, enhancing damage identification accuracy in real-world conditions.
- It offers a practical and scalable approach for intelligent structural health monitoring in real-world engineering applications.

**Abstract:**

Structural damage identification provides a theoretical foundation for the operational safety and preventive maintenance of in-service bridges. However, practical bridge health monitoring faces challenges in poor signal quality, difficulties in feature extraction, and insufficient damage classification accuracy. This study presents a bridge damage identification framework integrating time-varying filtering-based empirical mode decomposition (TVFEMD) with pre-trained convolutional neural networks (CNNs). The proposed method enhances the key frequency-domain features of signals and suppresses the interference of non-stationary noise on model training through adaptive denoising and time–frequency reconstruction. TVFEMD was demonstrated in numerical simulation experiments to have a better performance than the traditional EMD in terms of frequency separation and modal purity. Furthermore, the performances of three pre-trained CNN models were compared in damage classification tasks. The results indicate that ResNet-50 has the best optimal performance compared with the other networks, particularly exhibiting better adaptability and recognition accuracy when processing TVFEMD-denoised signals. In addition, the principal component analysis visualization results demonstrate that TVFEMD significantly improves the clustering and separability of feature data, providing clearer class boundaries and reducing feature overlap.

## 1. Introduction

Bridges are an essential component of modern transportation infrastructure, supporting heavy traffic flows and maintaining transportation connections between cities and regions. Bridge damage often progresses gradually, with initial damage being difficult to detect promptly through traditional manual inspections [1,2,3,4]. The expansion and progression of damage can significantly compromise bridge safety, potentially leading to catastrophic consequences [5,6,7,8]. In recent years, with the rapid advancements in sensor technology, signal processing, and artificial intelligence, vibration-based bridge health monitoring (SHM) has emerged as a mainstream monitoring approach [9,10,11,12]. Li et al. [13] proposed a novel damage index based on the monitoring of dynamic strain responses of steel beams under ambient vibrations before and after seismic events. Razavi et al. [14] conducted a comparative study on feature extraction methods based on dynamic response measurements, indicating that wavelet packet transform component energy (WPTCE) extracted from acceleration data exhibited a high sensitivity to structural damage. This method involves analyzing bridge vibration responses and employing algorithmic models to identify structural damage, thereby enabling a real-time monitoring of bridge health conditions. However, in practical monitoring scenarios, vibration signals are frequently influenced by environmental noise, traffic interference, and temperature fluctuations [15,16,17], resulting in degraded signal quality and presenting significant challenges for damage detection. Consequently, improving signal effectiveness in noisy environments and accurately extracting damage features remain critical challenges in current bridge health monitoring technologies.

In bridge health monitoring, signal denoising technology is a critical step in improving the accuracy of damage identification [18,19]. Existing signal denoising techniques can be categorized into frequency-domain methods and time-domain methods. Frequency-domain methods convert time-domain signals into frequency-domain signals using techniques such as Fourier transforms, remove specific frequency components, and then convert the signal back to the time domain to achieve denoising; examples include band-pass filtering and low-pass filtering. These methods typically assume that low-frequency components are useful information and high-frequency components are noise; however, this assumption does not always hold true in practice, limiting their denoising effectiveness. In contrast, time-domain denoising methods directly remove noise from the signal time history or reconstruct the signal by extracting useful components through multi-scale analysis. Common time-domain methods include Empirical Mode Decomposition (EMD) [20], Ensemble Empirical Mode Decomposition (EEMD) [21], and Wavelet Transform (WT) [22,23]. For instance, Wang et al. [24] proposed a damage identification method based on time-varying modal wavelet transform and successfully applied it to the damage detection of cantilever beams and large bridges. However, WT is sensitive to noise, which may affect identification accuracy. In comparison, the EMD is an adaptive signal decomposition technique, which handles nonlinear and non-stationary signals effectively [25]. Nevertheless, its recursive nature can lead to mode mixing, affecting the decomposition results.

The EMD and EEMD methods were subsequently developed to address these issues. For instance, Berrouche et al. [26] utilized EEMD for gear fault diagnosis and added white noise to reduce mode mixing. Although this method achieved good diagnostic results, the influence of noise was not completely eliminated. Most signal processing techniques based on decomposition, such as EMD and EEMD, face challenges such as mode mixing, endpoint effects, and waveform distortion [27], which may impact the extraction of damage features and identification accuracy. To overcome these limitations, the TVFEMD method was introduced, integrating time-varying filters into the EMD process, enabling real-time filtering during signal decomposition, and partially alleviating the aforementioned issues [28,29,30]. The effectiveness and reliability of TVFEMD in bridge measurement data have been validated, particularly in separating temperature effects, where TVFEMD significantly outperforms the traditional EMD and EEMD methods [31].

In the fields of image and pattern recognition, deep learning models, particularly convolutional neural networks (CNNs), have achieved significant success [32,33]. However, for directly applied to bridge damage detection, issues such as non-robust models, overfitting, and misclassification due to unreliable input signal quality are significant [34,35,36]. Therefore, it is essential to propose a new paradigm that integrates efficient signal reconstruction methods with deep feature extraction networks. Previous studies [37,38,39] have shown that combining VMD with shallow neural networks can enhance diagnostic performance, yet it still has limitations in removing high-frequency noise. In recent years, attention mechanisms, deep CNNs, and transfer learning techniques were introduced into the SHM domain [40,41]. However, most of these methods have overlooked the critical role of signal preprocessing at the input stage for deep models, resulting in significant performance fluctuations in complex signal-to-noise environments. Thus, it remains a pressing engineering challenge to integrate signal denoising with optimization of deep models to enhance their robustness and generalization capabilities effectively.

To the best of the authors’ knowledge, the TVFEMD method has been utilized in the fields of wind speed prediction and mechanical bearing fault diagnosis [42,43]. Zhang [44] proposed a denoising algorithm based on TVFEMD for fault classification of rotating machinery. Azimi [45] reviewed deep learning-based SHM models, discussing their capabilities and limitations. In practice, TVFEMD has performed in removing non-stationary noise, which is better for feature extraction structural damage scenarios. In addition, TVFEMD can perform processing of signals in both time and frequency domains through time-varying filtering, which provides better extracting features related to damage.

Therefore, this study develops a TVFEMD method integrating time-varying filters during the decomposition process to eliminate the need for forced upper and lower envelope symmetry. The challenging issues in traditional EMD, such as mode mixing and cumulative decomposition errors, were effectively addressed. After TVFEMD processing, the signals are encoded into two-dimensional images using MTF. The main innovations and contributions of this paper include: (1) proposing a signal denoising and feature extraction method based on TVFEMD, effectively suppressing complex noise and enhancing the prominence of damage features; (2) combining deep learning networks such as ResNet-50, EfficientNet-b0, and GoogLeNet to improve damage recognition accuracy and robustness while enhancing signal features; (3) verifying the advantages of the TVFEMD method in signal denoising and feature separation through PCA dimensionality reduction and visualization analysis. In the complex environment of actual engineering, this approach enhances the precision and stability of bridge damage detection.

The organization of the rest of the study is arranged as follows: Section 2 introduces the theoretical foundation of the TVFEMD method and presents the bridge damage detection framework combining TVFEMD with deep learning; Section 3 describes the experimental setup, scenario configuration, data collection, and signal processing; Section 4 discusses the findings, evaluating the effectiveness of this study by comparing different signal processing methods and deep learning models, and Section 5 is the conclusion and future prospects.

## 2. Methodologies

### 2.1. Time-Varying Filtering-Based Empirical Mode Decomposition

TVFEMD is used to separate multi-scale information from bridge monitoring data. The process is shown as follows:

(1) Determine the instantaneous amplitude *A*(*t*) and instantaneous phase *φ*(*t*) of the signal *x*(*t*) to be decomposed by using:

(1) $$A(t)=\sqrt{x{\left(t\right)}^{2}+\hat{x}{\left(t\right)}^{2}}$$

(2) $$\varphi (t)=\arctan\left(\frac{\hat{x}(t)}{x(t)}\right)$$

where the Hilbert transform of *x*(*t*) is denoted as $\hat{x}(t)$.

(2) By calculating the local maxima and minima of the instantaneous amplitude *A*(*t*), the corresponding analytical signal is defined as:

(3) $$z(t)=A(t)e^{j\varphi (t)}$$

where *z*(*t*) is the complex form of the signal, and *j* is the imaginary unit.

(3) Obtain the curves of the minimum and maximum values of *A*(*t*), which are used to calculate *β*_1_(*t*) and *β*_2_(*t*). Based on this, the instantaneous mean *a*_1_(*t*) and instantaneous envelope *a*_2_(*t*) can be calculated as:

(4) $$\left\{\begin{array}{l} a_{1}(t)=\left[\beta_{1}(t)+\beta_{2}(t)\right]/2\\ a_{2}(t)=\left[\beta_{2}(t)-\beta_{1}(t)\right]/2 \end{array}\right.$$

(4) Perform time-varying filter interpolation on *A*(*t_max_*) and *A*(*t_min_*) to obtain *η*_1_(*t*) and *η*_2_(*t*). The two IMF components *φ*_1_′(*t*) and *φ*_2_′(*t*) are then calculated by

(5) $$\left\{\begin{array}{l} \varphi_{1}^{\prime \;}(t)=\frac{\eta_{1}(t)}{2a_{1}^{2}(t)-2a_{1}(t)a_{2}(t)}+\frac{\eta_{2}(t)}{2a_{1}^{2}(t)+2a_{1}(t)a_{2}(t)}\\ \varphi_{2}^{\prime \;}(t)=\frac{\eta_{1}(t)}{2a_{2}^{2}(t)-2a_{1}(t)a_{2}(t)}+\frac{\eta_{2}(t)}{2a_{2}^{2}(t)+2a_{1}(t)a_{2}(t)} \end{array}\right.$$

(5) Compute the local cutoff frequency as:

(6) $$\varphi_{bis}^{\prime \;}\;(t)=\frac{\varphi_{1^{\prime }}(t)+\varphi_{2^{\prime }}(t)}{2}=\frac{\eta_{2}(t)-\eta_{1}(t)}{4a_{1}(t)a_{2}(t)}$$

(6) Use the time-variant filter to process the unfiltered signal and obtain the local mean. Calculate the signal *h*(*t*) and denote the final approximation result as *m(t)*:

(7) $$h(t)=\cos\left[\int \varphi_{bis}^{\prime \;}(t)dt\right]$$

(7) Assess whether the threshold criterion is satisfied to determine the existence of a narrowband signal. The selection of this threshold is based on the recommended values [46]. Narrowband signals have stable and clear frequency characteristics. If the criterion is met, the signal can be classified as a narrowband signal. Otherwise, update *x*(*t*) to *x*(*t*)-*m*(*t*), and repeat steps 1 to 6 until meeting the criterion:

(8) $$\theta (t)=\frac{B_{\mathrm{Loughlin}}(t)}{\varphi_{\mathrm{avg}}(t)}$$

where $B_{\mathrm{Loughlin}}(t)$ is the Loughlin instantaneous bandwidth, *ξ* is the bandwidth threshold, and *φ_avg_*(*t*) is the weighted mean instantaneous frequency. Continuously updating *x*(*t*), if the signal satisfies *θ*(*t*) ≤ *ξ*, then *x*(*t*) is considered as the IMF component at this time. The Loughlin instantaneous bandwidth and weighted average instantaneous frequency are written by

(9) $$B_{\mathrm{Loughlin}}{\left(t\right)}^{2}=\frac{a_{1}^{2}(t)+a_{2}^{2}(t)}{a_{1}^{2}(t)+a_{2}^{2}(t)}+\frac{a_{1}^{2}(t)a_{2}^{2}(t){\left(\varphi_{1}^{\prime \;}(t)-\varphi_{2}^{\prime \;}(t)\right)}^{2}}{{\left(a_{1}^{2}(t)+a_{2}^{2}(t)\right)}^{2}}$$

(10) $$\varphi_{\mathrm{avg}}(t)=\frac{a_{1}^{2}(t)\varphi_{1}^{\prime \;}+a_{2}^{2}(t)\varphi_{2}^{\prime \;}(t)}{a_{1}^{2}(t)+a_{2}^{2}(t)}$$

In SHM, the above methods can be used to effectively extract meaningful frequency characteristics from the vibration signals, thereby identifying damage. By updating *x*(*t*) and checking whether it meets the threshold conditions, characteristic representing bridge damage can be extracted iteratively. *x*(*t*) is the IMF signal sequence, and its frequency spectrum *X*(*f*) is obtained through the Fast Fourier Transform (FFT). Energy entropy is defined as:

(11) $$H_{X}(f)=-\sum_{i=1}^{N}\frac{|X(f_{i}){|}^{2}}{\sum_{i=1}^{N}|X(f_{i}){|}^{2}}\log\frac{|X(f_{i}){|}^{2}}{\sum_{i=1}^{N}|X(f_{i}){|}^{2}}$$

where $|X(f_{i}){|}^{2}$ represents the energy of the signal at that frequency point, and *N* is the total number of sampling points in the spectrum. The incremental IMF energy entropy ΔH quantifies the variation in energy entropy over time, representing the energy entropy increment in different iterations. The calculation formula is:

(12) $$\Delta H=H_{X}(f,t)-H_{X}(f,t-1)$$

where *H_X_*(*f*,*t*) is the energy entropy of the IMF signal at time t. By calculating the change in energy entropy Δ*H*, it is possible to assess whether the signal contains effective components. Effective components typically exhibit significant increases in energy entropy, while noise components exhibit smaller increases in energy entropy.

### 2.2. Deep CNNs

**Figure 1:**
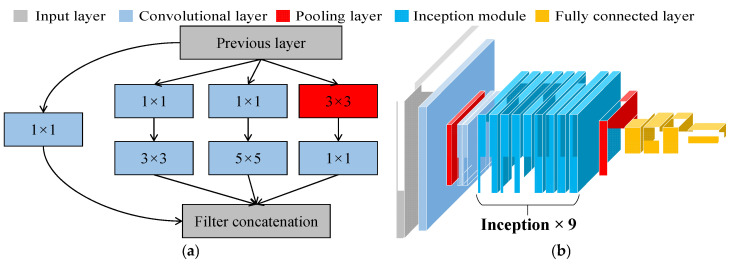
GoogLeNet network model: (**a**) Inception-v1 architecture diagram; (**b**) GoogLeNet network architecture.

With the rapid development of deep learning technology, CNNs have become core tools in signal processing and pattern recognition. In the field of bridge health monitoring and damage identification, CNNs have demonstrated exceptional performance. The GoogLeNet network [47] consists of 9 Inception modules connected in series. The Inception module effectively captures local features of different sizes by using 1 × 1, 3 × 3, and 5 × 5 convolution kernels in parallel, while the 3 × 3 pooling layer helps extract global information, as shown in Figure 1a. The Inception modules are capable of capturing features at different scales, enhancing the network ability to recognize various targets. Due to the introduction of the Inception structure, GoogLeNet has better performance compared with VGG and AlexNet in depth while having fewer parameters, resulting in lower memory and computational resource requirements. The overall structure of GoogLeNet is shown in Figure 1b, which includes an input layer, a 7 × 7 convolutional layer, a 3 × 3 max pooling layer, two 3 × 3 convolutional layers, and nine Inception modules. These components are connected to a global average pooling layer, followed by a Dropout layer, a fully connected layer, a Softmax layer, and the final classification output layer. This multi-scale feature fusion enables GoogLeNet to simultaneously focus on damage features at different levels and scales when processing bridge damage signals.

Deep residual networks [48,49,50] address the issues of vanishing and exploding gradients in deep networks by introducing residual connections, enabling effective training of deep network models without compromising performance. The residual modules in the ResNet50 network use skip connections to directly pass the input to subsequent layers of the network, avoiding signal loss in deep networks. The structure of each residual block in ResNet50 is as follows:

(13) $$y=F\left(x,\left\{W_{i}\right\}\right)-x$$

where $F\left(x,\left\{W_{i}\right\}\right)$ represents the feature maps obtained through convolution operations, written as follows:

(14) $$F\left(x,\left\{W_{i}\right\}\right)=\sigma \left(W_{2}\cdot \sigma \left(W_{1}\cdot x\right)\right)$$

where *W*_1_ and *W*_2_ are the weights of the convolutional layers, and σ represents the ReLu activation function.

To train the ResNet50 network, the cross-entropy loss function is typically used as the loss function, with the following formula:

(15) $$\mathrm{Loss}=-\sum_{i=1}^{C}y_{i}\log(p_{i})$$

where *y_i_* is the one-hot encoded true label, representing damage type *I*, and *P*(*i*) is the predicted probability of damage type *i* output by the network.

**Figure 2:**
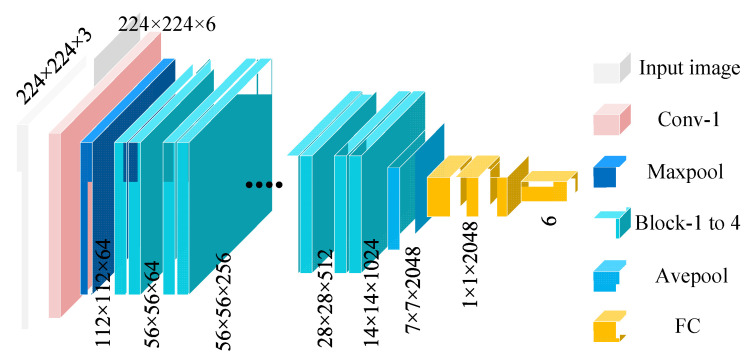
ResNet50 network architecture and residual block design.

Figure 2 shows the design of the ResNet50 network architecture. The residual network progressively extracts features through initial convolutional layers, pooling layers, and four residual modules (Block-1 to Block-4), and ultimately completes the classification task via a global average pooling layer (Avepool) and a fully connected layer (FC). To further enhance performance and training efficiency, ResNet leverages transfer learning strategies by utilizing models pretrained on the large-scale dataset ImageNet, transferring the learned general features to new tasks. The residual modules extract residual features from the input through convolutional layers and directly add these residuals to the input features via skip connections, generating the final output and ensuring effective transmission and reuse of features in deep networks.

**Figure 3:**
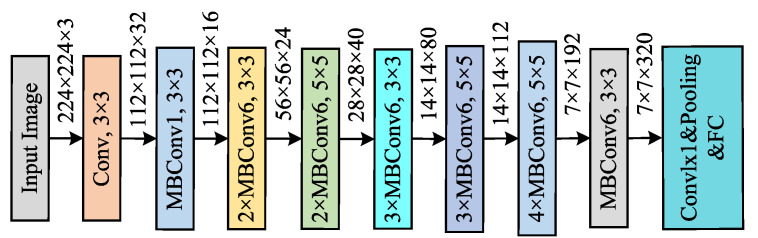
EfficientNet-B0 network architecture.

EfficientNet achieves a higher performance by balancing the width, the depth, and the resolution across three dimensions [51]. EfficientNet-b0 has fewer parameters and computational requirements but demonstrates an outstanding performance in multiple image classification tasks. Its innovation lies in optimizing the network’s scale through a compound scaling strategy, which reduces computational overhead while maintaining accuracy. The structure of the EfficientNet-B0 model is shown in Figure 3.

In bridge health monitoring, the efficiency of EfficientNet-b0 makes it an ideal choice [52], particularly in resource-constrained scenarios where it provides a high performance, complementing transfer learning to achieve a balance between computational efficiency and model performance. In terms of model parameters, EfficientNet-b0 significantly outperforms ResNet-50 with only 4.02 M parameters compared with 23.52 M for ResNet-50. This makes EfficientNet-b0 a more lightweight model, which is particularly advantageous in scenarios with limited resources. Although the average inference time per image for EfficientNet-b0 is 60.05 ms, slightly longer than ResNet-50’s 58.93 ms, the difference is minimal and can be considered negligible in many practical applications. Furthermore, due to its smaller parameter size, EfficientNet-b0 consumes less memory and computational resources, which is crucial for edge devices and low-power applications [53].

### 2.3. Markov Transition Field for Encoding Time Series

The Markov transition field (MTF) is an image encoding method designed to capture the dynamic characteristics of time series signals. It transforms one-dimensional time series data into two-dimensional feature images with temporal correlations, thereby preserving the state transition relationships within the time series in a visual form.

For a vibration time series signal *X* = {*x*_1_,*x*_2_,…,*x*_n_}, the signal is divided into *Q* quantile intervals based on its amplitude range. Each data point *x_i_* is assigned to the corresponding interval *q_j_* (where *j* ∈ [1,*Q*]). Based on the concept of a first-order Markov chain, the state transition probabilities between each pair of quantile intervals are written as follows:

(16) $$W=\left[\begin{array}{cccc} w_{11} & w_{12} & \ldots & w_{1Q}\\ w_{21} & w_{22} & \ldots & w_{2Q}\\ \vdots & \vdots & \ddots & \vdots \\ w_{Q1} & w_{Q2} & \ldots & w_{QQ} \end{array}\right]$$

(17) $$w_{ij}=p\left(x_{t}\in q_{i}|x_{t-1}\in q_{j}\right)$$

where *w_ij_* represents the probability of transitioning to state *q_i_* at the current moment given that the previous moment was in state *q_j_*.

Since the Markov chain is memoryless, the transition probabilities depend solely on the current state, thereby disregarding any dependencies or relationships that might exist over longer time spans. To overcome this shortcoming, we extend the traditional transition matrix *W* into a new matrix *M* that incorporates temporal position information. The matrix *M* is designed to account for not just the transition probabilities between the current state and the previous state, but also the effects of time evolution on these transitions.

(18) $$M=\left[\begin{array}{cccc} m_{11} & m_{12} & \ldots & m_{1N}\\ m_{21} & m_{22} & \ldots & m_{2N}\\ \vdots & \vdots & \ddots & \vdots \\ m_{N1} & m_{N2} & \ldots & m_{NN} \end{array}\right]=\left[\begin{array}{ccc} p\left(x_{1}\in q_{i}|x_{1}\in q_{j}\right) & \ldots & p\left(x_{1}\in q_{i}|x_{n}\in q_{j}\right)\\ p\left(x_{2}\in q_{i}|x_{1}\in q_{j}\right) & \ldots & p\left(x_{2}\in q_{i}|x_{n}\in q_{j}\right)\\ \vdots & \vdots & \vdots \\ p\left(x_{n}\in q_{i}|x_{1}\in q_{j}\right) & \ldots & p\left(x_{n}\in q_{i}|x_{n}\in q_{j}\right) \end{array}\right]$$

**Figure 4:**
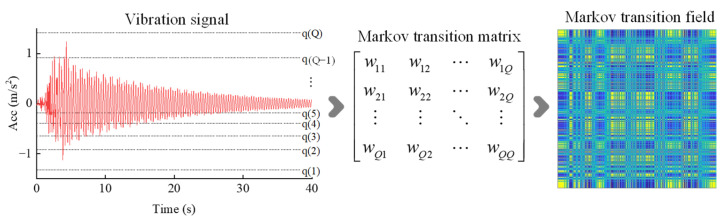
The Markov transition field for encoding time series signals.

The elements of the MTF matrix are mapped to color pixels, forming a two-dimensional image with temporal correlations, as shown in Figure 4. The patterns in the image can reflect differences between healthy and damaged states.

Unlike traditional frequency-domain techniques such as STFT and CWT, MTF, as a time-series encoding method based on state transition probabilities, better preserves global dynamic patterns. Preliminary experiments demonstrated that MTF encoding, when integrated with ResNet models, showed superior training stability and adaptability to small sample sizes, making it the preferred choice in this study.

### 2.4. A Bridge Damage Identification Framework Based on MTF

**Figure 5:**
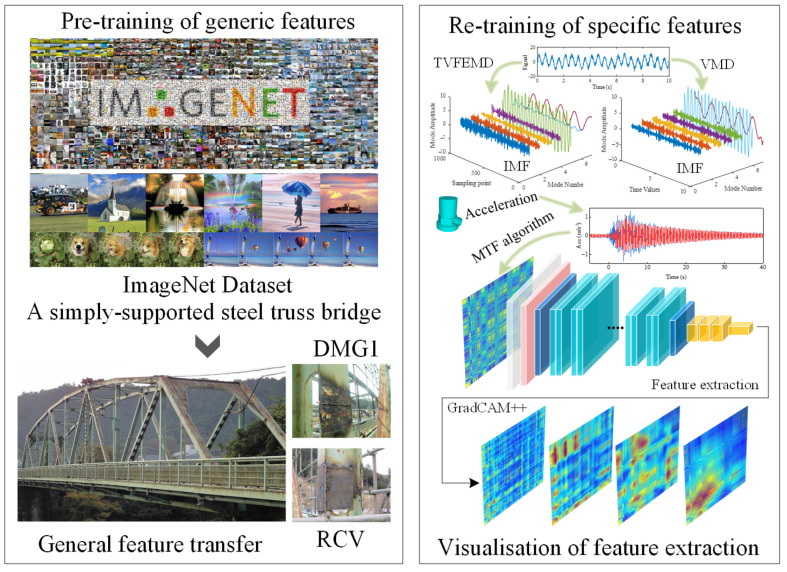
Bridge damage identification framework based on MTF.

The bridge damage identification based on MTF is shown in Figure 5. In the first part, to remove noise from bridge monitoring acceleration signals, this paper adopts the TVFEMD method. This method decomposes the original signal into multiple Intrinsic Mode Functions (IMFs) and selects low-frequency components for signal reconstruction, thereby obtaining denoised signals that accurately reflect the bridge’s health condition.

In the second part, the reconstructed acceleration signals are converted into two-dimensional images using the MTF method. The MTF calculates the transfer probabilities between signal time sequences, mapping the signal’s temporal dependencies into image features, which facilitates subsequent deep learning model identification.

In the third part, three deep learning networks, including GoogLeNet, ResNet50, and EfficientNet-b0, were utilized to perform damage identification on the converted images. GoogLeNet provides basic convolutional feature extraction, ResNet50 addresses the vanishing gradient problem in deep networks through residual learning, and EfficientNet-b0 optimizes computational efficiency through compound scaling. Model performance is evaluated using metrics such as accuracy, recall, and the F1 score. To demonstrate the capability of the CNN to automatically learn damage-related features from MTF-encoded inputs, GradCAM was employed to visualize the feature learning and classification mechanisms. GradCAM is a visualization technique for convolutional neural networks that generates heatmaps by computing the gradients of the class score with respect to the feature maps in the final convolutional layer, thereby highlighting the critical regions the model focuses on during prediction. As shown in the heatmaps of Figure 5, different regions of the input image contribute variably to the model’s classification, with the gradient-based color intensities indicating the level of contribution.

This framework integrates TVFEMD signal processing, MTF image conversion, and deep learning to efficiently extract damage features from bridge monitoring data. By comparing the performance of different networks, the optimal model is selected, demonstrating strong practical application potential, particularly in SHM.

## 3. Case Study

### 3.1. Numerical Signal

**Figure 6:**
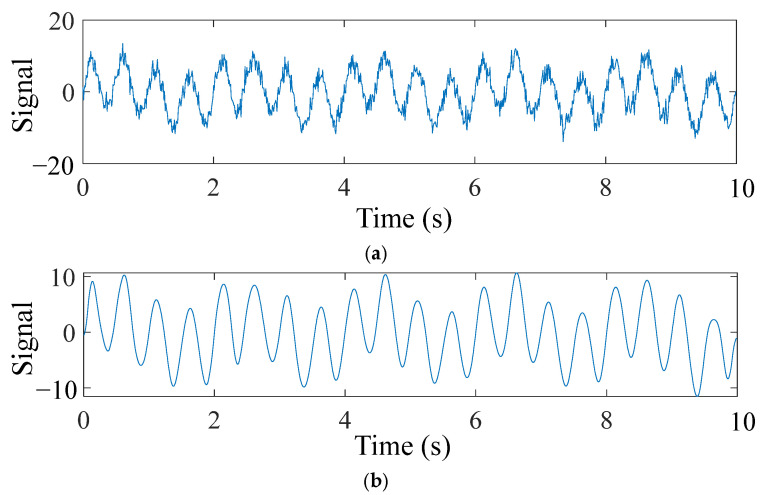
Numerical signals before and after TVFEMD denoising and reconstruction: (**a**) Noisy signal; (**b**) Denoised signal.

Bridge monitoring data often comes with noise at different frequencies. Therefore, a simulated signal was constructed using two sine signals, *y*_1_ and *y*_2,_ with frequencies of 0.5 Hz and 2 Hz, respectively, and a Gaussian white noise *y*_3_ with a signal-to-noise ratio of 10 dB. The signal parameters are: *y*_1_ = 3sin (2 × 0.5πt); *y*_2_ = 7sin (2 × 2πt). The sampling frequency is 100 Hz, and the duration is 10 s. The original signal and the signal after TVFEMD denoising are shown in Figure 6.

**Figure 7:**
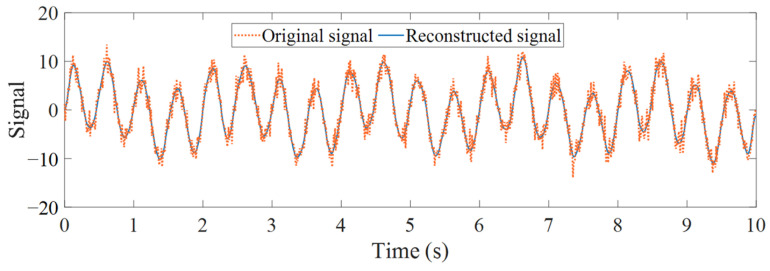
Comparison of VMD denoising and reconstruction.

The numerical signal after VMD denoising and reconstruction is shown in Figure 7. To verify the effectiveness and superiority of the proposed method, both the TVFEMD and VMD methods were used to decompose the simulated signal. The bandwidth threshold and B-spline order parameters for TVFEMD were set to 0.25 and 26, respectively [48].

**Figure 8:**
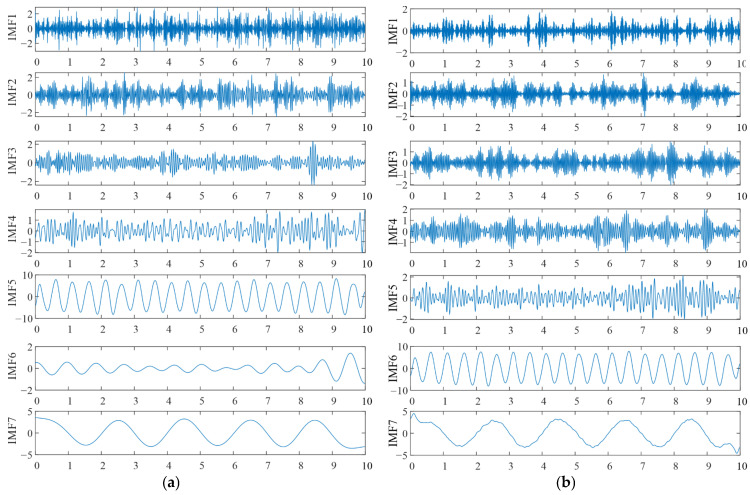
Decomposition results of two methods: (**a**) TVFEM; (**b**) VMD.

Figure 8 shows the decomposition results of the two methods. It can be seen that both methods successfully extracted the signals of different frequencies. The low-frequency components obtained by TVFEMD are smoother, while VMD exhibited mode mixing in the low-frequency components, where similar characteristics were distributed across different components at different time scales. Compared to VMD, TVFEMD can more precisely separate components with similar frequencies, especially in lower-order IMFs. This demonstrates TVFEMD’s advantages in maintaining modal purity and enhancing the interpretability of time-frequency features, which is crucial for subsequent structural damage assessment.

**Figure 9:**
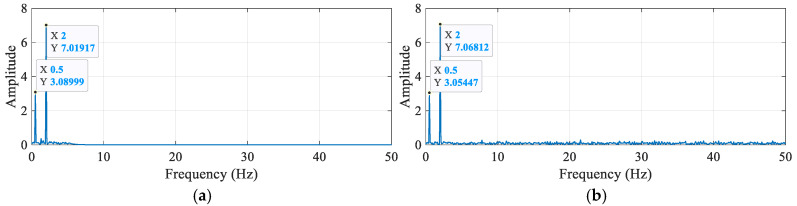
Comparison of effective IMF component spectra under different methods: (**a**) IMF component spectrum using TVFEMD; (**b**) IMF component spectrum using VMD.

By performing a fast Fourier transform (FFT) on the IMF energy entropy increment, the true components were extracted, and spectral analysis was conducted. Figure 9 shows the effective component spectra under different methods. The results confirm that the signal frequencies are 0.5 Hz and 2.0 Hz, validating that the IMF energy entropy increment can accurately select these effective signals. The amplitudes of the simulated signals are 3 mm and 7 mm, and the effective signal amplitudes obtained using TVFEMD are more precise than those obtained using VMD. Additionally, compared with VMD, TVFEMD exhibits lower spectral energy in non-main frequency bands, indicating its superior ability to suppress frequency noise while retaining effective components.

### 3.2. The Old ADA Bridge

**Figure 10:**
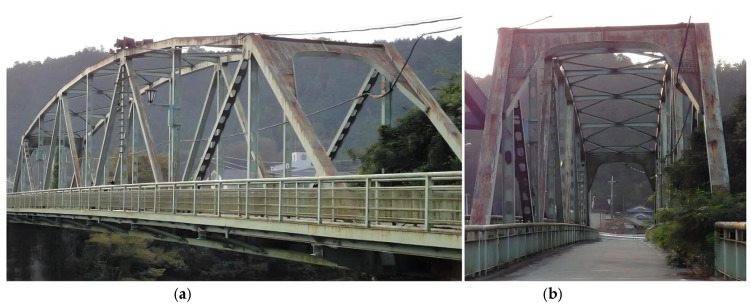
Old ADA Bridge: (**a**) elevation view; (**b**) cross-sectional view.

**Figure 11:**
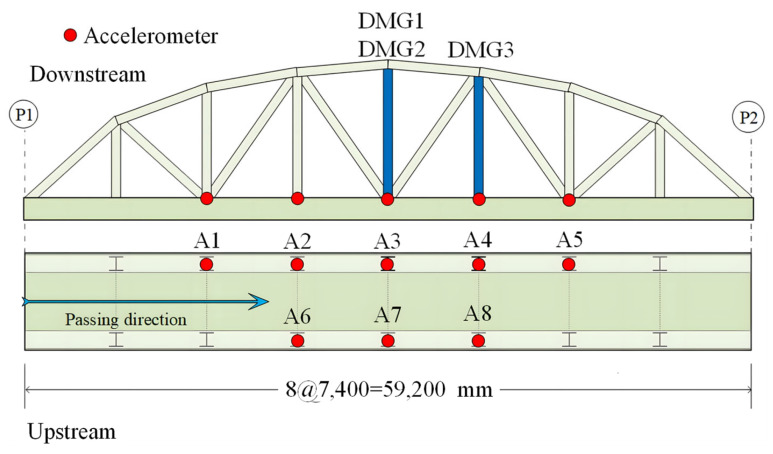
Layout of accelerometer sensors.

**Figure 12:**
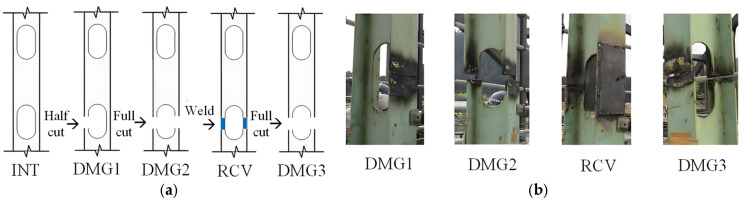
Schematic diagram and photographs of damage modes for the Old ADA bridge: (**a**) damage mode schematic diagram; (**b**) photograph of damaged locations. (The blue color indicates repaired components).

The Old ADA Bridge is located in Nara Prefecture, Japan, as shown in Figure 10. It is a simple-span steel truss bridge with a main span of 59.2 m and a bridge width of 3.6 m [54]. The bridge was demolished in 2012. Prior to its demolition, field vibration tests were conducted on the bridge under undamaged and four different damage conditions. The layout of the accelerometer measurement points is shown in Figure 11, with a sampling frequency of 200 Hz. The specific damaged locations are shown in Figure 12 [55]. It can be observed that five sensors were installed on one side of the damaged truss components, and three additional sensors were installed on the other side, providing sufficient information for bridge damage identification. This section investigates bridge damage identification using a CNN under moving vehicle loading.

This study used data from all eight sensors. Under undamaged and four different damage conditions, 160 samples were collected for each condition, totaling 800 samples. The data were obtained from the vertical acceleration responses at eight measurement points on the deck of the Old ADA Bridge in Japan, acquired under vehicle excitation. The test details are shown in Table 1. During the testing process, the vibration responses of the deck under different damage conditions were recorded. The measured dataset was constructed using a sliding window method, with a sample size of 4500 × 2 × 10 × 8 for each damage condition. The dataset was divided into training, validation, and test sets in a 3:1:1 ratio. The training and validation sets were used to train the model, while the test set was used to evaluate the model’s performance in damage identification.

**Figure 13:**
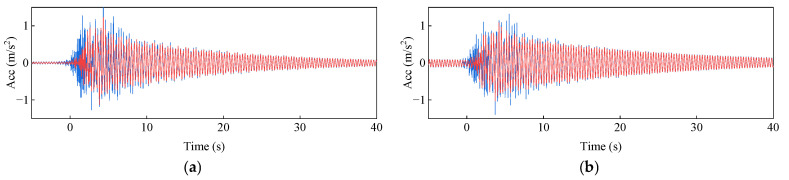
Comparison of signals before and after TVFEMD processing: (**a**) INT; (**b**) DMG1.

**Figure 14:**
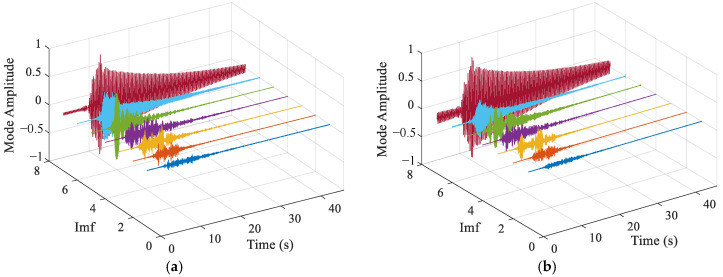
Decomposition results of TVFEMD: (**a**) INT; (**b**) DMG1.

To improve signal quality and eliminate interference, appropriate signal processing techniques were applied to denoise and reconstruct the original data. Figure 13 shows a comparison of reconstructed signals under healthy and damage modes. The blue waveform represents the original signal, while the red waveform represents the reconstructed signal. It can be observed from Figure 13 that the reconstructed signal retains the main features of the original waveform and reduces high-frequency noise components, thereby enhancing the signal-to-noise ratio. To analyze the intrinsic components of the signal, TVFEMD was employed for signal decomposition. Figure 14 presents the decomposition results for healthy and damage modes. The decomposed IMFs are displayed in the form of three-dimensional surface plots. TVFEMD effectively decomposes the signal into multiple IMFs, capturing different oscillation patterns. The TVFEMD method can finely separate components with similar frequencies during the signal decomposition process.

## 4. Results and Discussion

### 4.1. Hyperparameter Settings and Training Processes

In this experiment, the hyperparameters for the bridge damage detection framework based on CNNs were set as follows: the initial learning rate was set to 0.0001, with a low learning rate chosen to avoid rapid convergence to a suboptimal solution during the early stages of training while ensuring training stability. The learning rate for the classifier was set to 0.001, the momentum coefficient for Stochastic Gradient Descent (SGD) was set to 0.9, the weight decay value was set to 0.01, and the number of training iterations was set to 150. The ResNet-50 architecture was developed using the PyTorch 1.12.1 framework and trained and tested on a computer equipped with an Intel(R) Core i9-13700 CPU and an NVIDIA GeForce RTX 4070 GPU.

**Figure 15:**
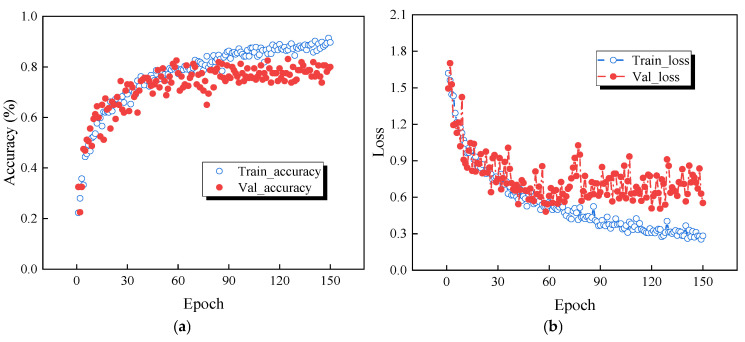

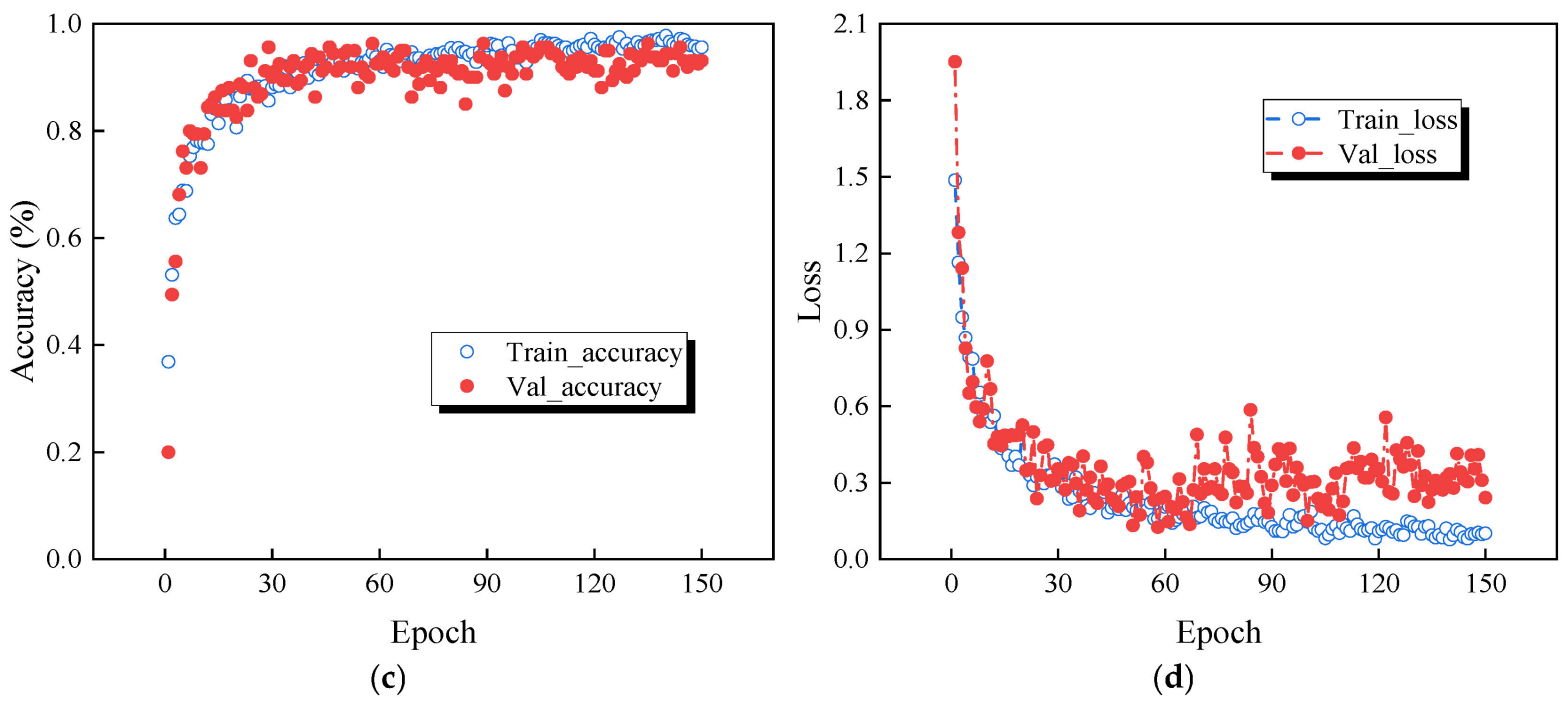
Accuracy and loss comparison of the ResNet-50 network: (**a**) accuracy of the original signal; (**b**) loss of the original signal; (**c**) accuracy of TVFEMD reconstructed signal; (**d**) loss of TVFEMD reconstructed signal.

The training process was conducted using the ResNet-50 network, chosen for its deep residual learning capability and strong generalization ability. Figure 15 shows the variation in accuracy and loss of the original signal and the TVFEMD denoise-deconstructed signal during training. Figure 15a,b represents the training accuracy and loss for the original signal, where blue circles indicate the training set and red circles indicate the validation set. It can be observed that the validation accuracy of the original signal gradually increases with the number of training epochs and stabilizes around the 60th epoch. However, the validation loss for the original signal remains at a high level throughout, and in the later stages of training, both the validation accuracy and loss exhibit significant fluctuations, indicating instability in the model’s generalization performance on the validation set and potential overfitting.

Figure 15c,d shows the accuracy and loss for damage identification using the TVFEMD denoise-deconstructed signal. After TVFEMD processing, both the training and validation accuracy exhibit a smoother upward trend, with a more noticeable improvement in validation accuracy. The loss also shows lower fluctuations and gradually stabilizes and decreases, suggesting that TVFEMD effectively enhances the model’s stability and recognition accuracy after signal denoising and reconstruction. Compared with the original signal, the TVFEMD denoised and reconstructed signal demonstrates more stable and superior performance in terms of accuracy and loss, validating the effectiveness of TVFEMD in improving damage identification tasks.

### 4.2. Comparison of Different Signal Processing Methods

**Figure 16:**
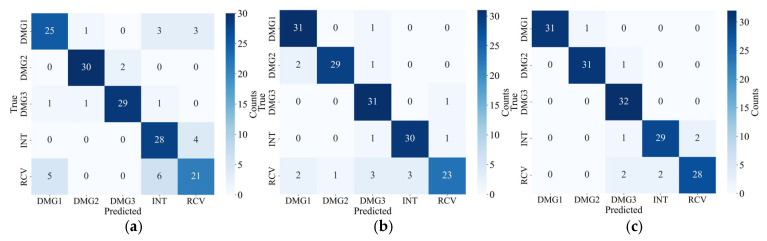
Confusion matrix of different signal processing methods: (**a**) original; (**b**) VMD; (**c**) TVFEMD.

Figure 16 presents the confusion matrix for damage identification using ResNet-50, comparing the effects of different signal processing methods on the identification results. Specifically, Figure 16 corresponds to the following three signal processing methods: original signals without any processing; signals denoised and reconstructed using VMD; and signals denoised and reconstructed using TVFEMD. As shown in Figure 16, the damage identification results for original signals exhibit significant confusion among different categories, particularly between the INT and RCV categories, with a noticeable number of misclassifications by the classifier. Specifically, the RCV category in original signals is frequently misclassified as DMG1 and INT, indicating that unprocessed signals are significantly affected by noise interference, which impairs the accurate identification of damage patterns.

In contrast, signals processed with VMD demonstrate higher accuracy in damage identification. Notably, in the identification of the DMG1 category, the classifier can better distinguish between different damage states, with a marked reduction in misclassifications. However, signals processed with VMD still exhibit some misclassifications, particularly in the identification of the RCV category, where misclassification into other categories has not been eliminated. Signals processed with TVFEMD achieve the best recognition performance. Under this processing method, the misclassification rate in the confusion matrix is significantly reduced, with a substantial improvement in the identification accuracy of the RCV category. Through its multi-scale adaptive filtering, TVFEMD can effectively remove noise and avoid overlapping with the main frequency components of the signal. As shown in Figure 16, between INT (intact) and RCV (repaired), the signal characteristics are inherently weak due to their similar physical states. Once affected by noise interference, the model is prone to confusion. TVFEMD enhances the detailed differences between these two types of signals, significantly reducing the misclassification rate in the confusion matrix.

**Figure 17:**
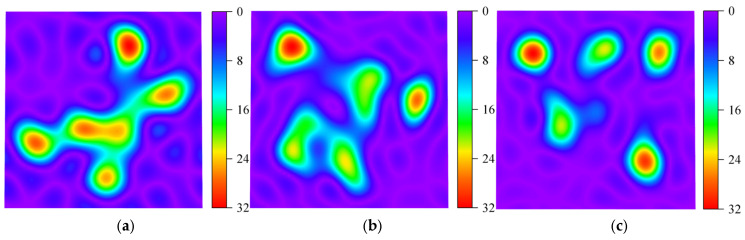
T-SNE feature distribution heatmap: (**a**) original; (**b**) VMD; (**c**) TVFEMD.

Figure 17 presents a feature distribution heatmap generated using the T-SNE method to visualize the effects of different signal decomposition approaches. Figure 17a shows the damage-sensitive features of the original signal, where the patterns are relatively complex, lacking clear separability, and the clustering boundaries are blurred. As seen in Figure 17b, the VMD method effectively separates the signal features, though some areas remain somewhat mixed, failing to fully decouple different frequency components. The TVFEMD method achieves clearer separation of frequency components, resulting in purer signal modes and a more pronounced clustering of damage features, as illustrated in Figure 17c.

### 4.3. Comparison of Different CNNs

**Figure 18:**
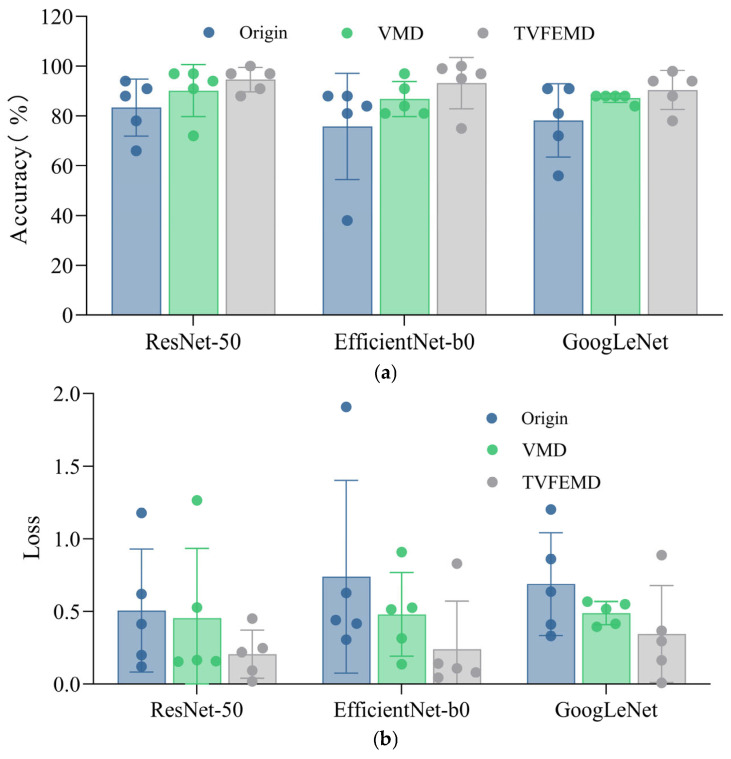
Comparison of accuracy and loss among the three networks: (**a**) accuracy; (**b**) loss.

Figure 18 presents a comparison of damage localization effects among three different networks when subjected to different signal processing methods. The comparison is evaluated using two metrics: accuracy and loss. TVFEMD can accurately separate different frequency components of signals in the time-frequency domain, effectively removing noise and interference. As shown in Figure 18a, the accuracy comparison reveals that signals processed by TVFEMD exhibit the best recognition performance across all network architectures. Specifically, the accuracy rates for TVFEMD-processed signals in ResNet-50, EfficientNet-b0, and GoogLeNet networks are 94%, 93%, and 90%, respectively, which are significantly higher than those of the original signals. The accuracy of VMD denoising reconstructed signals also improves compared with the original signals, particularly in ResNet-50 and GoogLeNet architectures, reaching 90% and 87%, respectively. However, these results still fall short of those obtained from TVFEMD-processed signals. As seen in Figure 18b, TVFEMD-processed signals demonstrate the lowest loss values across all network architectures. Signals reconstructed via VMD also show some improvement in loss values.

In addition to accuracy, recall and the F1 score are commonly used as important metrics to evaluate the classification performance of each category [56]. To comprehensively assess the classification effectiveness of various models, this study also compares the performance of different pre-trained networks using recall and the F1 score. Recall represents the ratio of correctly predicted positive samples to the actual positive samples:

(19) $$\;\mathrm{Recall}\;=\frac{TP_{i}}{TP_{i}+FN_{i}}$$

where TP denotes the number of samples correctly classified into the corresponding category, and FN denotes the number of samples that do not belong to the category but were incorrectly classified into it. The F1 score is the harmonic mean of precision and recall, taking into account both the accuracy and completeness of the classification model. Its value ranges from 0 to 1, with 1 indicating the best classification performance. When precision and recall differ significantly, the F1 score tends to shift toward the lower value. Its calculation formula is as follows:

(20) $$F_{1}\mathrm{Score}=\frac{2\cdot \frac{1}{N}\sum_{i=1}^{N}\frac{TP_{i}}{TP_{i}+FP_{i}}\cdot \frac{1}{N_{\mathrm{cl}}}\sum_{i=1}^{N}\frac{TP_{i}}{TP_{i}+FN_{i}}}{\frac{1}{N}\sum_{i=1}^{N}\frac{TP_{i}}{TP_{i}+FP_{i}}+\frac{1}{N}\sum_{i=1}^{N}\frac{TP_{i}}{TP_{i}+FN_{i}}}$$

where TP is the number of true positive predictions, FP is the number of false positive predictions, and FN is the number of false negative predictions.

**Figure 19:**
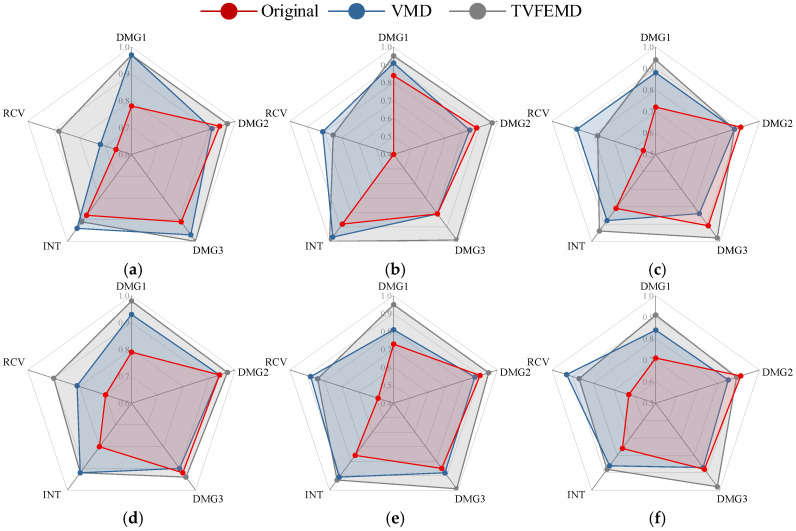
Comparison of classification performance for different combinations of networks and denoising methods: (**a**) ResNet-Recall; (**b**) Efficientnet-b0- Recall; (**c**) GoogLeNet- Recall; (**d**) ResNet- F1 score; (**e**) Efficientnet-b0- F1 score; (**f**) GoogLeNet- F1 score.

Figure 19 shows the impact of different signal processing methods on the classification performance of pre-trained networks, using Recall and the F1 score as evaluation metrics. It comprehensively compares the performance of three typical deep neural networks—ResNet-50, EfficientNet-b0, and GoogLeNet—in damage identification tasks, presented in a radar chart format. The aim is to systematically evaluate the recognition capabilities of various models under different signal processing conditions, providing a reference for selecting a more optimal network structure in engineering applications. As shown in Figure 19, signals reconstructed after TVFEMD processing achieved the best classification performance across all network architectures, in terms of both recall and the F1 score. Particularly in the identification of damage categories such as DMG1 and RCV, TVFEMD processing significantly improved recall and resulted in more stable and reliable classifier outputs. In comparison, while the VMD method improved noise interference in the original signals to some extent, its performance across most categories still fell short of TVFEMD. The original signals performed the worst across all evaluation metrics. In comparisons among different network architectures, the pre-trained ResNet-50 demonstrates the best overall performance across all three signal processing conditions, making it the preferred model for engineering applications. EfficientNet-b0 follows closely, while GoogLeNet shows a relatively weaker performance in terms of recall and the F1 score, indicating certain limitations in extracting complex damage features.

### 4.4. Feature Extraction Visualization

**Figure 20:**
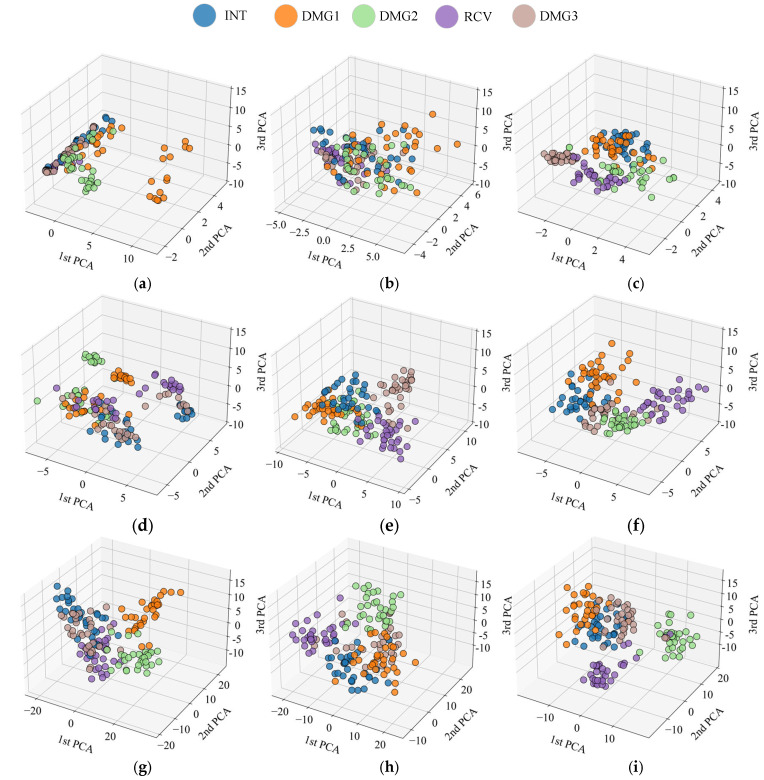
Comparison of data feature distributions extracted by PCA: (**a**) GoogLeNet-original; (**b**) GoogLeNet-VMD; (**c**) GoogLeNet-TVFEMD; (**d**) Efficientnet-b0- original; (**e**) Efficientnet-b0-VMD; (**f**) Efficientnet-b0-TVFEMD; (**g**) ResNet50- original; (**h**) ResNet50-VMD; (**i**) ResNet50-TVFEMD.

Figure 20 shows the distribution of high-dimensional features from three pre-trained networks after dimensionality reduction to a 3D space using PCA. PCA, including a linear dimensionality reduction tool, extracts the directions of maximum variance, thereby filtering out some noise, focusing on core information, and enabling the structural features of high-dimensional data to be presented in a 3D space. Figure 20a,d,g represents the feature points of the raw signal, which show a significantly mixed distribution across the three network architectures, with blurred the boundaries between categories. This indicates that the raw input still carries a significant amount of noise, which affects the expression of high-level features. Figure 20b,e,h shows that after VMD processing, the trend of feature aggregation is enhanced, noise interference is somewhat alleviated, and the spatial discriminability between categories is improved, particularly evident in EfficientNet-b0 and ResNet-50. This suggests that VMD is beneficial for denoising frequency band information. Figure 20c,f,i demonstrates that after TVFEMD processing, the feature point clouds exhibit the most distinct clustering effect across all networks, with clear boundaries between categories. This indicates that this method significantly improves feature separability by more effectively removing noise and reconstructing signal structures.

It can be seen from Figure 20 that the deep learning models with signal reconstruction demonstrate superior performances in feature extraction. Furthermore, by comparing network architectures, ResNet-50 network demonstrated the clearest feature distribution among all processing methods, and category clustering was further enhanced after TVFEMD processing. The EfficientNet-b0 network performed slightly worse than ResNet-50, but after TVFEMD processing, it also demonstrated strong feature separability and high accuracy, indicating that this method has a significant impact on lighter networks as well. Although the feature distribution of GoogLeNet improved after TVFEMD processing, its robustness to noise was poor, resulting in relatively blurred category boundaries. Nevertheless, TVFEMD still improved its classification performance to some extent.

## 5. Conclusions

In practical bridge engineering, structural health monitoring faces challenges in low signal quality, difficulty in feature extraction, and insufficient accuracy in structural damage classification. This study proposed a damage identification framework that combines TVFEMD with a pre-trained convolutional neural network. By enhancing signal feature separability and suppressing redundant noise, this method significantly improves classification performance and model stability. The main conclusions are summarized as follows:

(1) The TVFEMD method is introduced into deep learning-driven structural damage identification tasks, enabling adaptive denoising and time-frequency reconstruction of raw sensor signals. Compared with the traditional VMD method, TVFEMD can effectively suppress the interference of non-stationary noise in model training while preserving critical damage features.

(2) The ResNet-50 model has the strongest adaptability and highest recognition performance to features processed by TVFEMD, compared with GoogLeNet and EfficientNet-b0 models. Therefore, ResNet-50 is more suitable for bridge damage identification tasks, offering better stability and engineering applicability.

(3) Under raw signal conditions, certain micro-damage categories are difficult to distinguish in the feature space. PCA provides interpretability for the deep network’s recognition mechanism, and the results show that TVFEMD-processed data exhibits better clustering and clearer class boundaries in the feature space, effectively mitigating the issue of feature overlap in raw signals and providing more discriminative input.

This study provides a feasible and highly effective solution for deep learning-based structural damage identification tasks. However, challenges remain in practical engineering deployment, such as model light-weighting, real-time responsiveness, and cross-scenario adaptability. In further study, a lightweight TVFEMD feature extraction mechanism will be developed for edge-side signal preprocessing. In addition, multi-source heterogeneous sensor data (e.g., strain, images, acoustic emissions) will be integrated to enhance the model performance.

## Figures and Tables

**Table 1:** Damage modes of the old ADA bridge damage cases.

Damage Scenario	Description
INT	Full bridge intact
DMG1	Half cut in a vertical member at midspan
DMG2	Full cut in a vertical member at midspan
RCV	Recovery of the cut member at midspan
DMG3	Full cut in a vertical member at 5/8th-span

## Data Availability

Data will be made available on request.
